# Physicochemical and functional characterization of gelatin edible film incorporated with fucoidan isolated from *Sargassum tenerrimum*

**DOI:** 10.1002/fsn3.3402

**Published:** 2023-05-03

**Authors:** Mohsen Pouralkhas, Moazemeh Kordjazi, Seyed Mahdi Ojagh, Omid Asadi Farsani

**Affiliations:** ^1^ Department of Fisheries, Faculty of Fisheries and the Environment Gorgan University of Agricultural Sciences and Natural Resources Gorgan Iran

**Keywords:** antibacterial characteristics, biodegradable film, fucoidan, gelatin, mechanical properties

## Abstract

Biodegradable films were created with fish gelatin and fucoidan extracted from *Sargassum tenerrimum* using 30% glycerol as a plasticizer. The gelatin films were incorporated with fucoidan (2.5%, 5%, 7.5%, and 10%), respectively. Results presented that the average thickness of films ranged from 0.12 to 0.147 mm. Tensile strength (TS) was decreased from 29.27 to 3.46 MPa by adding the fucoidan except for the gelatin/fucoidan 10% (5.35 MPa) sample. The results showed that the physical characteristics (the contact angle (Ɵ), water solubility, opacity, and moisture content) of the films significantly changed depending on different fucoidan concentrations. FTIR and SEM analysis confirmed the interaction of fucoidan with gelatin in the composite film. Furthermore, adding 10% fucoidan showed high DPPH radical scavenging activity (65%) than other treatments. Therefore, incorporation of fucoidan extracted from brown algae (*Sargassum tenerrimum*) with fish gelatin films improved thermal stability, anti‐oxidative, and antibacterial characteristics in addition to enhanced mechanical and protective properties, to be used as a bioactive edible film in the food packaging industry.

## Graphical abstract

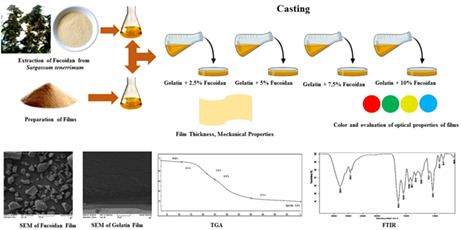

## 1 INTRODUCTION

In the last decade, synthetic plastics are widely used in the food industry as packaging polymers, but the use of these polymers due to their very slow biodegradation has caused many environmental problems (Jouki et al., 2013). Since microbial contamination is one of the main reasons for spoilage and deterioration of food quality, film production has attracted a lot of attention in the food packaging industry (Clarke et al., 2017). The use of edible and biodegradable films has been considered one of the active alternative packaging methods for nondestructible plastic films (Rui et al., 2017). Oral films maintain the quality and shelf life of food by controlling the transfer of moisture, carbon dioxide, oxygen, and food additives (Arfat et al., 2014).

Gelatin is a protein obtained from the hydrolysis of collagen in the skin, bones, and connective tissue of animals, including livestock, poultry, and aquatic animals. Fish gelatin is also a good source of biopolymers for film packaging. It is biodegradable and has good film properties (Etxabide et al., 2017). In recent years, due to the prevalence of bovine disease and the rejection of pig gelatin by religious groups, research on other raw sources of gelatin has increased (Nagarajan et al., 2017). Gelatin can be extracted from fishery waste such as bone and fish skin. The physical and chemical properties of fish gelatin, including gel resistance, melting and boiling temperatures, and rheological properties are weaker than those of mammalian gelatin (Sow et al., 2018). Studies on film production from fish gelatin have shown that gelatin produced from fish also has excellent film‐forming properties (Qiao et al., 2017).

Gelatin, with its unique properties, has many applications in the food, pharmaceutical, medical and photographic industries, including the ability to produce a gel, its adhesion, clarity, and thickening properties (Das et al., 2017).

Lipid oxidation is a major contributor to reducing the quality of foods by negatively affecting their sensory properties, shelf‐life, and nutritional value. To prevent such oxidative damage, synthetic antioxidants are most commonly used as food additives. The toxicity of synthetic antioxidants butylhydroxyanisole (BHA) and butylhydroxytoluene (BHT) have paved the way for the search for unique natural antioxidants (Wang et al., 2022).

Gelatin‐based packaging films can be used as an antimicrobial and antioxidant agent to improve the quality and shelf life of packaged foods (Benbettaïeb et al., 2019). On the other hand, seaweed is a rich source of bioactive compounds that can produce many types of metabolites with a wide range of biological activities. Compounds with antioxidant, antiviral, and antimicrobial activity have been identified in brown, red, and green algae (Cox et al., 2010). Seaweeds are limited due to their abundance as a suitable alternative to terrestrial resources (Khalil et al., 2017). One of their bioactive compounds is fucoidan, which is obtained from brown algae and is a polysaccharide (Fawzy et al., 2017). Applicable properties of fucoidan include film formation, antimicrobial, antioxidant, biodegradable, anticoagulant, anti‐inflammatory, and wound healing properties and are used in the medical industry (Gomaa et al., 2018; Hifney et al., 2017; Perumal et al., 2018). Sulfated fucoidan derived from brown seaweeds such as Porphyra, *Laminaria japonica*, *Undaria pinnatifida*, *Sargassum polycystum*, *Kjellmaniella crassifoliacan* can be as a potential source of natural antioxidants, as it has excellent free radical scavenging, hydroxyl radical scavenging activity, superoxide anion radical scavenging, and ferric reducing power activities (Xu & Wu, 2021). Packaging with antioxidant releasing system is a kind of food preservation method, in which an antioxidant incorporated into the package will increase the shelf‐life of foods (Govindaswamy et al., 2018). Fish gelatin being a carrier of functional components, it has been proposed to combine fucoidan derived from brown seaweed in gelatin to produce an edible film. This research has been considered because no research has been done on the gelatin/fucoidan film extracted from Persian Gulf algae.

## 2 MATERIALS AND METHODS

### 2.1 Chemicals

Calcium chloride, 2,2‐Diphenyl‐1‐picrylhydrazyl (DPPH), 2,2′‐Azino‐bis (3‐ethylbenthiazoline‐6‐sulfonic acid) diammonium salts (ABTS), and glycerol were purchased from Merck company, gelatin from cold water fish skin, solid was obtained from Sigma‐Aldrich. Other chemicals such as ethanol and acetone were obtained from Merck company.

### 2.2 Extraction of fucoidan from *Sargassum tenerrimum*

Brown algae (*Sargassum tenerrimum*) were collected from the Persian Gulf (Iran) and used for the extraction of fucoidan. Algae were first washed with seawater and twice in sterile distilled water to remove sediment and foreign substances; then, air dried under shade and powdered using an electric mill. Dried algae powder (20 g) was treated with 80% ethanol (200 mL) at room temperature in a magnetic stirrer to remove proteins and pigments; then washed with acetone and centrifuged at 5000× *g* for 10 min. Dried algae were extracted by distilled water (1:20 w/v) at 65°C with shaking and centrifuged at 5000× *g* for 10 min at 4°C. The collected supernatant was concentrated and mixed with 1% calcium chloride (1:1 v/v) solution and placed at 4°C overnight to precipitate alginate. After centrifugation, the supernatant was collected again and treated with ethanol (30%), and kept overnight at 4°C. The supernatant was again mixed with 70% ethanol overnight at 4°C and centrifuged again to precipitate the fucoidan. Intact fucoidan was washed with ethanol and acetone and finally dried at room temperature (Govindaswamy et al., 2018).

### 2.3 Preparation of gelatin–fucoidan films

Lyophilized gelatin 4% (w/v) was dissolved in distilled water under continuous stirring in a magnetic stirrer at room temperature for 15 min. Glycerol (30% w/w) was added as a plasticizer and stirred for 10 min. The film‐forming solutions were made by adding 0 (control gelatin), 2.5%, 5%, 7.5%, and 10% of fucoidan. Solutions were homogenized for 30 min using a high‐speed magnetic stirrer, and then 15 mL of it was poured into circular plastic plates at 25°C and dried for 24 h. The dried films were then manually separated and used for further analysis.

### 2.4 Physical and mechanical properties of films

#### 2.4.1 Film thickness

A digital micrometer (with an accuracy of 0.001 mm, Mitutoyo Corporation) was used to measure the thickness of the films. Measurements were repeated randomly at five points in each film. The average thickness of these points was used to determine the physical and mechanical properties of the films (Gibis et al., 2012).

#### 2.4.2 Mechanical properties

Tensile strength (TS) and elongation at break (E%) of films were measured using Instron Universal Testing Machine (Model A1 700, Gotech) using method number d882‐01 approved by ASTM (ASTM, 2002). The films were cut into rectangles measuring 2.5 × 10 cm^2^ and stored in a desiccator containing magnesium nitrate (to create a relative humidity of 53%) at 25°C for 48 h. The samples were then placed between the two jaws of the device. The initial distance between the two jaws and the movement speed of the upper jaw were determined to be 50 mm and 50 mm/min, respectively, and the data were recorded by a computer (Ghanbarzadeh & Almasi, 2011). The tensile strength of the films was calculated from the following equation:

#### 2.4.3 Water solubility

The water solubility of gelatin‐fucoidan films was measured according to Jeya Shakila et al. (2012). The films (2.5 × 2.5 cm) were first dried in an oven at 105°C for 24 h to obtain a constant initial weight. The dried samples were then placed in containers containing 50 mL of distilled water for 24 h. they were filtered through Whatman filter paper no. 1. The residue separated from the filter paper and weighed after drying in an oven at 105°C for 24 h. The solubility of the samples was calculated according to the following formula:

$$\text{Solubility}\;\left(\%\right)=\frac{w0-\mathrm{wf}}{w0.}$$

where Wo: initial weight of the sample, Wf; weight of the undissolved desiccated residue.

#### 2.4.4 The contact angle (Ɵ)

The contact angle of the films was measured by the sessile drop method, which is a common method for determining the hydrophobicity of solid surfaces. For this purpose, 5 μL of deionized water was placed on the samples and the contact angle of the drop with the film was reported at the initial time. In this test, 5 replications were considered for each sample.

#### 2.4.5 Color and evaluation of optical properties of films

Colorimeter (BYK Gardner Model) was used to determine the color of the prepared films. The films were placed on a standard white screen (L* = 94/63, a* = −0 / 88, b* = 0.65). The color parameters such as L* (lightness/brightness), a* (redness/greenness), and b* (yellowness/blueness) values were recorded by 5 replicates. ∆E (total color difference values of the film were calculated) as reported by Lanier et al. (1991).

$$\mathrm{\Delta E}=\sqrt{{\left(∆L^{*}\right)}^{2}+{\left(∆a^{*}\right)}^{2}+{\left(∆b^{*}\right)}^{2}}$$

#### 2.4.6 Opacity

The light transmission spectrum of the samples was measured at 200–800 nm by a spectrophotometer (Lambda 25, Perkine Elmer) in 5 replications. The device was calibrated with an empty tube. Then the samples (1 × 4 cm^2^) were pasted into the tube and placed in a spectrophotometer. The following formula was also used to calculate the opacity of the films (Abdollahi et al., 2013):

$$\text{Opacity}\;\left(O\right)=\mathrm{Abs}600∕x$$

where Abs 600 = value of absorbance at 600 nm, ‘*x*’ = the film thickness (mm).

### 2.5 Scanning electron microscopy (SEM)

Morphology of films was displayed using a scanning electron microscope (Philips XL30, Netherlands). Films were cut into 3 × 3 cm and glued to an aluminum base with silver glue. The bases were coated with gold in a coating/spraying apparatus. The surface of the samples was imaged at different magnifications.

### 2.6 Fourier transform infrared spectroscopy

Fourier transform infrared spectroscopy (FTIR) spectra of films were determined using FTIR Spectrometer (Perkin‐Elmer, Spectrum Rxi) in the range of 400 to 4000 cm^−1^ and in the resolution of 4 cm^−1^.

### 2.7 Thermal gravimetric analysis (TGA)

The dried films were analyzed. from 25 to 600°C with a heating rate of 10°C/min in a nitrogen gas at a flow rate of 20 mL/min using TGA‐7 (Perkin Elmer).

### 2.8 Antioxidative activities of gelatin–fucoidan films

DPPH radical scavenging activity of films was determined according to the method presented by Govindaswamy et al. (2018). Film solution was prepared by solubilizing the treatments in distilled water to obtain a 5 mg/mL protein concentration. Then, methanolic DPPH solution (1 mL, 0.1 mM) was added to each treatment, mixed well, and placed for 2 h in the dark at room temperature. The absorbance was read at 517 nm using a UV–Vis spectrophotometer (Shimadzu UVmini‐1240) distilled water was used as control. DPPH radical scavenging activity was measured based on the following formula:

$$\text{DPPH radical scavenging activity}\;\left(\%\right)=\left(1-\frac{\mathrm{Abs}\;\text{sample}}{\mathrm{Abs}\;\text{control}}\times 100\right)$$

### 2.9 Microbial analysis

Three circular pieces were cut from each film (1 cm in diameter). *Staphylococcus aureus* (ATCC 29737) and *Escherichia coli* (ATCC 10536) strains were obtained from the microbial collection of the University of Tehran (Iran). Some of each bacterium was removed using a sterile loop from sterile ampoules and added to 10 mL of BHI Broth culture medium. The culture medium was incubated for 24 h at 37°C. The tubes were linearly cultured on a Nutrient Agar medium using a sterile loop. The plates were autoclaved and incubated for 24 h at 37°C. 3–5 well‐isolated colonies were transferred to tubes containing 5 mL of physiological saline. The turbidity of the suspensions was examined by a spectrophotometer at 625 nm. Each tube was suspended on its culture medium. Then, the films were cut into circular disks (10 mm in diameter). the films were placed in a culture medium inoculated with the suspension at appropriate intervals, and the plates were incubated at 37°C for 24 h. Then inhibition zone diameter around the film was reported in millimeters.

### 2.10 Statistical analysis

All experiments were done in triplicates and the results are indicated as an average mean ± SD. A significant difference between the means was defined by Duncan's multiple comparison test using the statistical software Statistical Package for the Social Sciences Version 26 (SPSS Inc.).

## 3 RESULT AND DISCUSSION

### 3.1 Physical properties (solubility, moisture, contact angle, film thickness)

The results of the physical properties of the prepared films are presented in Table 1. Results showed that the moisture content of films significantly increased with the addition of fucoidan from 12.02% in the gelatin treatment to 20.80% in the treatment containing 10% fucoidan. The moisture content represents the total volume occupied by the film network microstructure by water molecules, while the solubility index is related to the hydrophilicity of the films (Jiang et al., 2010). The lower amounts of moisture in gelatin films compared to gelatin–fucoidan films are probably due to the reduction of capacity and voids in the polymer substrate. This result also indicates that gelatin showed more hydrophobic properties than protein and fucoidan films. Solubility is one of the important properties of biodegradable films that can determine the degree of resistance of the film to water, especially in humid environments such as meat foods (Gimenez et al., 2009).

**TABLE 1:** Thickness, moisture, water solubility, and Contact angle of gelatin–fucoidan films.

Treatment	Moisture (%)	Water solubility (%)	Contact angle (°)	Film thickness (mm)
Gelatin	12.02 ± 0.3c	92.63 ± 0.85a	91.11 ± 1.09a	0.133 ± 0.0ab
Gelatin:Fucoidan 2.5%	17.70 ± 0.9b	91.12 ± 0.31b	89.47 ± 0.46a	0.147 ± 0.02a
Gelatin:Fucoidan 5%	18.79 ± 0.56b	90.15 ± 0.76b	89 ± 1.23a	0.147 ± 0.02a
Gelatin:Fucoidan 7.5%	20.57 ± 0.93a	88.50 ± 0.75c	87.90 ± 1.24a	0.120 ± 0.01b
Gelatin:Fucoidan 10%	20.80 ± 1.41a	71.68 ± 0.08d	88.68 ± 1.38a	0.147 ± 0.03a

*Note*: Different lowercase superscripts in the same column indicate a significant difference (*p* ≤ .05), Mean ± SD (*n* = 3).

The average water solubility of gelatin film was expressed (92.63%) which agreed with the similar previous study reported for the cod gelatin films (88%), catfish (83.3%), and swim bladder of rohu (*Labeo rohita*) (91.49%; Hoque et al., 2010). Our results displayed that the incorporation of fucoidan in gelatin films had decreased the water solubility from 92.63% to 71.68%.

The water solubility of gelatin–fucoidan films significantly increased (P < 0.05) with the incorporation of fucoidan (2.5%, 5%, 7.5%, and 10%) which could be due to the formation of more interactions between biopolymers, leading to lower availability of hydroxyl groups to absorb the water molecules. Earlier, Hosseini et al. (2013) reported similar results for the edible film produced by gelatin and chitosan.

In another research, using seaweed (*Turbinaria ornata*) extract incorporated with fish gelatin films reported reduced water solubility (Rattaya et al., 2009). Cross‐linking of gelatin composite film with other biopolymers decreases low molecular fractions, which reduce the water solubility (Wang et al., 2022).

The fucoidan polysaccharide cross‐links by electrostatic interactions with low molecular fractions of gelatin, which stabilizes the gelatin–fucoidan film matrix, and thus limits the water solubility. The results of the contact angle of the smooth surface of produced films showed that the composite films containing fucoidan did not display a significant difference compared to the control film, but by increasing in fucoidan level, the contact angle of the composite films decreased somewhat. This is probably due to hydrophilic groups at the biopolymer level.

Thickness is one of the most important parameters used to evaluate the mechanical properties and opacity of films. The mean thickness of edible films ranged from 0.12 to 0.147 mm (Table 2) and it was increased with the addition of fucoidan significantly (*p* ≤ 0.05). A similar trend in the thickness of fish gelatin films in combination with polysaccharides and carrageenan (Pranoto et al., 2007) chitosan, and essential oil (Wu et al., 2015) was declared by some researchers. The addition of other macromolecules such as polysaccharides, film preparation method, and drying process are factors influencing the thickness of gelatin‐based films. Our results are in agreement with other studies (Hoque et al., 2010; Govindaswamy et al., 2018; Lupina et al., 2022; Ratna Aprilia et al., 2022). They found that a low increase in the thickness is usually due to the protruding construction formed during film formation by gelatin strands. The destruction of compact network and reduction in the regular alignment of gelatin chains might cause the protruded structure. Fucoidan is a polysaccharide compound with different molecular compositions that can uniquely interact with gelatin and form different alignments in the composite film matrix, which in turn contributes to increasing the film thickness.

**TABLE 2:** Color analysis and opacity of gelatin–fucoidan films.

Treatment	L*	a*	b*	Opacity
Gelatin	93.47 ± 1.17^a^	2.97 ± 0.23^c^	00.00 ± 0.00^c^	34.92 ± 1.42e
Gelatin:Fucoidan 2.5%	92.80 ± 0.95^a^	1.60 ± 1.39^c^	7.03 ± 4.75^b^	39.04 ± 1.37^d^
Gelatin:Fucoidan 5%	86.83 ± 1.15^b^	3.93 ± 2.08^bc^	24.03 ± 0.92a	43.38 ± 1.05^c^
Gelatin:Fucoidan 7.5%	87.50 ± 5.67^b^	6.27 ± 1.55^b^	24.83 ± 0.92a	48.46 ± 1.28b
Gelatin:Fucoidan 10%	76.57 ± 2.15^c^	12.27 ± 1.17^a^	25.90 ± 0.00a	53.38 ± 0.75^a^

*Note*: Different lowercase superscripts in the same column indicate a significant difference (*p* ≤ .05), Mean ± SD (*n* = 3).

### 3.2 Film color and opacity

Film color is an important factor because of its direct effect on the appearance of packaged products and consumer acceptance (Bortom et al., 2008). Transparency of films is an important feature of food packaging in terms of its direct relationship with the appearance of product (Abdullah et al., 2022). The greater the transparency of biodegradable films and similar to synthetic polymers, the greater the use of this type of packaging material. The transparency of the films can affect the oxidation rate of fats and the quality of the packaged product (Abdullah et al., 2022). Results showed that the lightness (L*) value of films was higher in gelatin films, (Table 2), which reduced the gelatin–fucoidan films from 93.47 in gelatin film to 76.57 in gelatin–fucoidan 10%, significantly (*p* ≤ .05). Besides, redness (a*) and yellowness (b*) values increased in gelatin–fucoidan films (*p* < .05) with increasing fucoidan concentration.

Similar results were also considered by Peng and Li (2014) in the chitosan‐based film using thyme, lemon, and cinnamon essential oil. Furthermore, the incorporation of 2% gellan and k‐carrageenan to fish gelatin film significantly decreased lightness (L*) as the films, which were darker than the control treatment, whereas a* and b* values did not reduce significantly (Said et al., 2021).

Materials used in the packaging industry must be able to protect food from the effects of light, especially UV radiation. Protein‐based films have a great UV barrier property due to the presence of high amounts of aromatic amino acids that absorb UV light (Ningruma et al., 2021). However, the plasticizers used in the film formulation compete with the polymers for hydrogen bonds, disrupting the homogeneity of the network and forming transparent films (Begines et al., 2022).

The opacity of gelatin–fucoidan films varied with adding the fucoidan (Table 2). Gomez‐Estaca et al. (2009) found that the addition of plant extracts to the gelatin film although protecting the transparency, turned the films darker and affected the light transmission. Our results showed that gelatin film incorporated with 10% fucoidan had higher opacity values (53.38%) than other treatments, as noticed in a previous study (Rattaya et al., 2009). The changes in transparency in gelatin–fucoidan films were because of the formation of cross‐links among the molecules that form the new structures, as well as to the scattering of light by the fucoxanthin molecules in the film network via the electrostatic interaction or hydrogen bonding.

### 3.3 Mechanical Properties

Mechanical properties of prepared films are studied to evaluate the film stiffness, stretching ability, and film strength. The results of mechanical properties are presented in Table 3. Adding the fucoidan to the gelatin film resulted in significant changes in its mechanical properties so that the tensile strength (TS) of films was initially decreased by adding the fucoidan and the highest amount was observed in the gelatin film treatment (29.27 MPa).

**TABLE 3:** Mechanical properties of gelatin–fucoidan films.

Treatment	Tensile strength (MPa)	Elongation at Break (%)
Gelatin	29.27 ± 0.01^a^	47.31 ± 13.04^a^
Gelatin:Fucoidan 2.5%	4.32 ± 0.01^c^	58.53 ± 4.33^a^
Gelatin:Fucoidan 5%	3.46 ± 0.0^e^	38.30 ± 15.07^a^
Gelatin:Fucoidan 7.5%	3.93 ± 0.01^d^	46.90 ± 26.23^a^
Gelatin:Fucoidan 10%	5.35 ± 0.01^b^	62.47 ± 15^a^

*Note*: Different lowercase superscripts in the same column indicate a significant difference (*p* ≤ .05), Mean ± SD (*n* = 3).

The TS of gelatin–fucoidan films decreases by adding fucoidan at low levels and then increased by increasing fucoidan (7.5 and 10%) from 3.93 to 5.35 MPa (*p* ≤ .05) (Table 3).

In agreement with this study, an increase in TS with incorporating polysaccharides to fish gelatin film (Pranoto et al., 2007); from 8 to 23 MPa by adding the chitosan in skin gelatin film were observed (Gomez‐ Estaca & Lopez de Lacey, 2010). Factors such as hydrophobicity, surface charge, and polymer chain length are the agents affecting the mechanical properties of protein layers. Hydrogen bonds in the layers mainly improve TS protein films. Moreover, the properties of gelatin‐based film layers strongly depend on the softeners and film thickness (Wang et al., 2022). The results showed that the strength and flexibility of layers will change with changes in the amounts and ratio of protein to polysaccharides. Pranoto et al. (2007) found that the optimal level of gelatin and polysaccharide for better interaction between them was 2%. In our study, the composition of gelatin‐fucoidan 10% was prepared as a compact film with better tensile properties. The average EAB values of gelatin–fucoidan films increased from 47.31% to 62.47% with the increase in fucoidan concentration (Table 3). It is important to study the different mechanical properties of films because of the need for edible polymers to have the desired strength and elasticity and to be free from defects such as cavities and small fractures.

### 3.4 Film microstructure (SEM)

**FIGURE 1:**
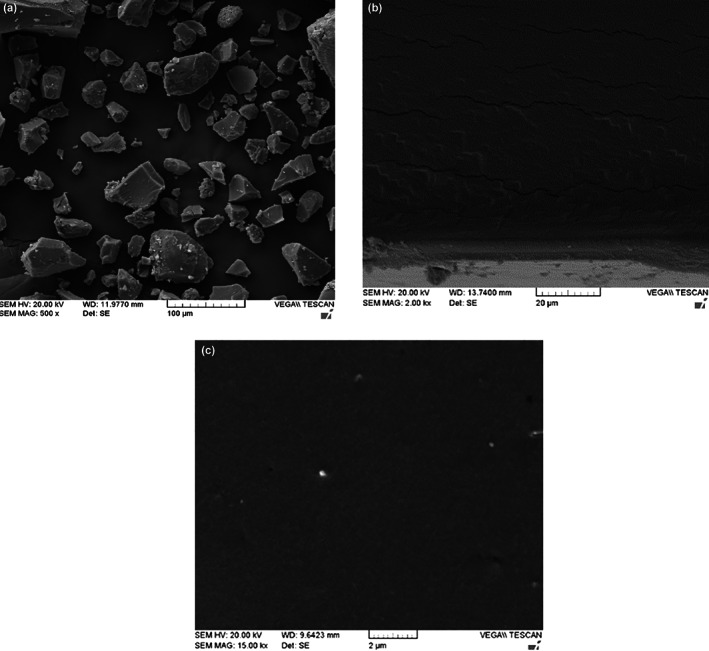
SEM image of the morphology of fucoidan (a), gelatin (b), and gelatin–fucoidan 5% (c) films.

Figure 1 indicates the microstructures of gelatin–fucoidan composite films given by SEM. The surface of pure gelatin film represents compact film with no pores. However, a discontinuous zone specified by horizontal direction cracks is randomly distributed along networks in gelatin films. By adding the fucoidan, the cross‐section was smooth, uniform, and without cracks and pores and visible insoluble particles which showed the uniform distribution of polysaccharides in the biopolymer substrate, network formation and establishment of bonds between fucoidan and myofibril proteins. Pranoto et al. (2007) found that the incorporation of gellan also improves the internal structure to give the compact and dense appearance, however, this initiated some linkages and resided between fibrillar zones of fish gelatin through polyelectrolyte association.

### 3.5 FTIR spectroscopy analysis

FTIR analysis of fucoidan showed the primary absorptive peaks were associated with glycosidic structures which were attributed to C‐O and C‐C stretching vibrations of the pyrenoid ring and the C‐H group. The FTIR spectrum showed a peak at 3423 cm^*−*1^ which was related to a hydroxyl stretching vibration. Another peak at 2925 was consistent with the stretching vibration of the C‐H bond of the pyrenoid ring. Previous studies showed that the wide signal at 1250 cm^−1^ represents the total sulfate esters in the polysaccharides (Barros et al., 2013; Synytsya et al., 2010).

**FIGURE 2:**
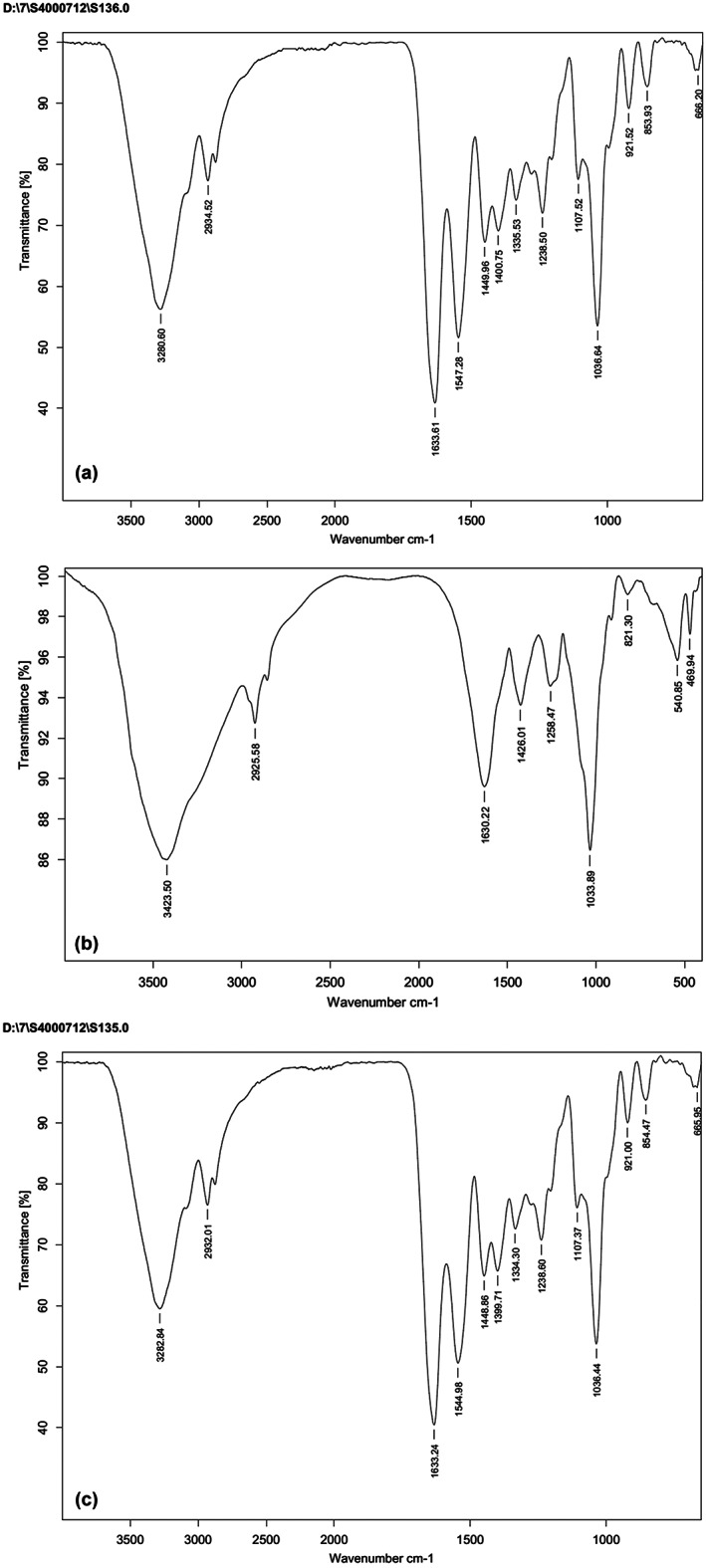
FTIR spectra of gelatin (a), fucoidan (b) and gelatin– fucoidan 5% (c) films.

To examine the functional groups, structure, and interactions of gelatin with other molecules, FTIR spectra of gelatin–fucoidan films are analyzed (Said et al., 2021). In this study, amide‐A‐related peaks at 3280 and 3282 cm^−1^ were discovered in gelatin and gelatin–fucoidan films (Figure 2).

These peaks correlate to NH stretching and hydrogen bonding. Moreover, in FTIR analysis of gelatin‐based films, amide B peak at 29332–2934 cm^−1^ approves C–H stretching and –NH3+, which is specific to gelatin. Similarity, amide B peaks were considered at 2928 cm − 1 in skin fish gelatin film incorporated with herbal extract (Nagarajan et al., 2013; Tongnuanchan et al., 2013). Using fucoidan in film formulation had shifted this peak to a lower wave number (2932 cm^−1^) because of asymmetrical stretching vibration of C–H in the CH2 group. Amide I peak was observed at 1633 cm^−1^, which links to C=O stretching and hydrogen bonding coupled with COO− (Aewsiri et al., 2009). A peak was like the present study related to amide I was reported in the tilapia skin gelatin layer made with k‐carrageenan and gellan at 1650 cm^−1^. Amide III peak at 1238 cm^−1^ was created due to vibrations in the C–N and N–H groups of bound amide or vibration of CH_2_ groups of glycine. Similarly, the peaks that occurred at 921 cm^−1^ were characteristic of gelatin molecules.

### 3.6 Thermal gravimetric analysis (TGA) of prepared films

**FIGURE 3:**
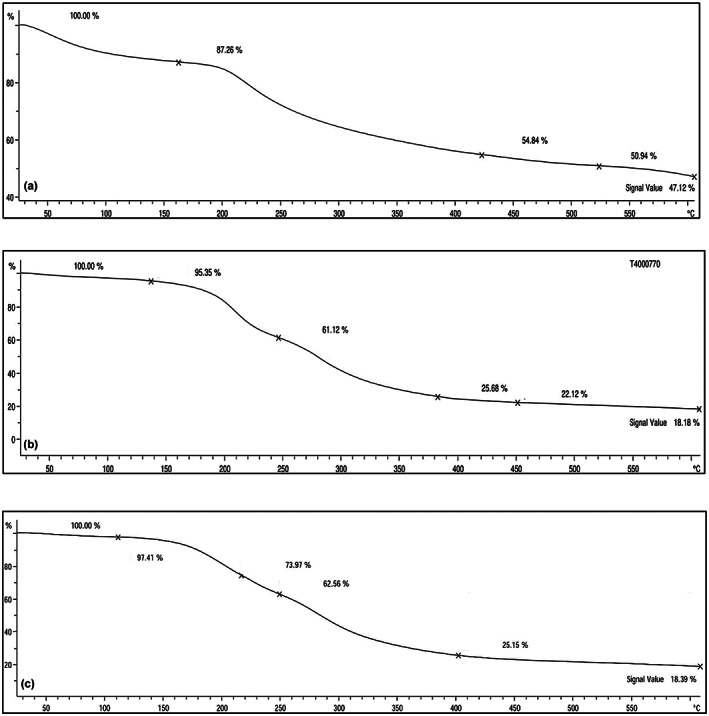
TGA curves for: (a) Gelatin, (b) Fucoidan, (c) Gelatin:Fucoidan 5%.

Thermal gravimetric analysis curves shown in Figure 3 represented four main stages of decomposition and weight loss in all films. The initial weight loss observed in all films at lower temperatures is probably due to the reduction or evaporation of free water, bound water, and also volatile compounds absorbed in the films. According to Figure 3, in a fucoidan film, the first step involves the removal of physically absorbed water, while the second stage deals with the removal of chemisorbed water concurrently with the destruction of the fucoidan molecule. The destruction of the polysaccharide macromolecule is followed by stages three and four (Matusiak et al., 2022).

Fucoidan film began to degrade thermally around 520°C as a result of the random breakage of glycosidic linkages. The remaining mass at the end of this process (600°C) was changed into ash, which contains minerals recognized in fucoidan like phosphates, carbonates, and sulfates (Saravana et al., 2018). In gelatin film (Figure 3b) the most important temperature for the beginning of weight loss is the second stage of degradation, which indicates the beginning of thermal degradation and decomposition of films, which is attributed to the degradation or decomposition of parts of protein with low molecular weight and structural bonds related to water. Figure 3b,c showed the gelatin: fucoidan composite curve determined it starts to decline earlier than the native gelatin. Lower weight loss in composite films can be related to lower moisture content than in pure gelatin film. Composite films showed higher thermal stability in stages 2 and 3 of degradation compared with gelatin film. The highest residual weight percentage was observed in the fucoidan film and this indicated the higher thermal stability of the gelatin: fucoidan composite gel. This result indicated that adding the fucoidan at appropriate levels can improve the thermal stability of gelatin biofilm. According to Wang et al. (2019), the addition of biopolymers can improve the thermal stability of gelatin‐based films. In complexes, there are electrostatic interactions that present enhanced thermal stability. Previous studies showed that gelatin‐based film formulations containing natural phenolic compounds can form a strong gelatin network with the phenolic molecules, able to raise the denaturation temperature and increasing the thermal durability of the films (Ratna Aprilia et al., 2022; Wang et al., 2019).

### 3.7 Antioxidative activities of gelatin–fucoidan films

DPPH free radical scavenging test was used to estimate the antioxidant capacity of the fucoidan, gelatin, and gelatin–fucoidan films. Previous findings reported that the antioxidant capacity of fucoidan is due to its ability to donate H atoms to the free radical 2,2‐diphenyl‐1‐picrylhydrazyl (purple) resulting in 2,2‐diphenyl‐1‐picrylhydrazyl (yellow; Wang et al., 2022).

DPPH radical scavenging potential of the fucoidan extracted from *Sargassum tenerrimum* had 89% activity at a 10 mg/mL concentration (Raghu et al., 2016). Alboofetileh et al. (2019) reported that three factors like chemical structure, phenol, and sulfate contents, are capable of affecting fucoidan compounds' bioactivity. Besides the fucoidan compounds, brown seaweed has other secondary metabolite compounds, namely, phenolics, flavonoids, alkaloids, glycosides, tannins, and steroids, which are believed to have antioxidant activity. In this work, gelatin film did not display any DPPH radical scavenging activity (Table 4), as reported by several authors (Abdullah et al., 2022; Gimenez et al., 2009; Said et al., 2021; Tongnuanchan et al., 2014). However, the composite films (gelatin‐fucoidan 10%) had the maximum DPPH radical scavenging activity of 65%. Similar results indicated that the addition of brown algae extracts (*Cystoseira barbata*) to fish gelatin film had improved the DPPH's radical scavenging potential (Govindaswamy et al., 2018). In agreement with our study, incorporating essential oils in gelatin films also enhanced the DPPH radical scavenging activity from 25% to 100%.

**TABLE 4:** Antioxidant activity of gelatin–fucoidan films.

Treatment	DPPH radical scavenging (%)	Total antioxidant
Gelatin	‐	0.21 ± 0.00^e^
Gelatin:Fucoidan 2.5%	50 ± 13.101 ^d^	0.48 ± 0.01^d^
Gelatin:Fucoidan 5%	53.233 ± 6.101^c^	0.68 ± 0.00^c^
Gelatin:Fucoidan 7.5%	62 ± 13.101^b^	0.96 ± 0. 01^b^
Gelatin:Fucoidan 10%	65 ± 36.102^a^	1.43 ± 0.01^a^

*Note*: Different lowercase superscripts in the same column indicate a significant difference (*p* ≤ .05), Mean ± SD (*n* = 3).

### 3.8 Antimicrobial properties of prepared films

The antimicrobial activity of gelatin film as well as gelatin‐fucoidan films with different concentrations against *Staphylococcus aureus* and *Escherichia coli* were evaluated and compared with co‐amoxiclav, gentamicin, and tetracycline antibiotics (Table 5). The results showed that gelatin films without fucoidan did not have any antimicrobial properties but films containing fucoidan exhibited significant antimicrobial activity. Antibacterial activity against the studied bacteria was significantly varied by changing the level of fucoidan in the film. Previous studies have noticed that gram‐positive bacteria were more sensitive to seaweed extracts as compared to gram‐negative bacteria (Ayrapetyan et al., 2021) which was in agreement with the results obtained in our research. Ayrapetyan et al. (2021) and Palanisamy et al. (2019) reported that fucoidan inhibited growth in *Staphylococcus aureus* and *Escherichia coli* microorganisms, and showed a bacteriostatic effect against both gram‐positive and gram‐negative bacteria. Two common reasons for the antibacterial mechanisms of polysaccharides have been suggested. It can be explained that sulfated polysaccharides attach to the surface of bacteria and thus cause damage and leakage of nutrients. This hypothesis has been substantiated by identifying the nucleic acids and proteins (Wang et al., 2022) released after the treatment of microorganisms with sulfated polysaccharides. Another hypothesis is the antibacterial effect of fucoidans by trapping nutrients. Cationic minerals, for example, are explained in the nutrient medium by negatively charged molecules of sulfated polysaccharides, which leads to reduced bioavailability of nutrients to microorganisms. Therefore, according to the results, it can be seen that the bacteriostatic effect on the studied bacteria is due to fucoidan, which prevents the bacteria from receiving adequate nutrition, which leads to inhibition of bacteria (Ayrapetyan et al., 2021).

**TABLE 5:** The inhibition zone (diameter in mm) produced by gelatin‐based biofilm with 2.5%, 5%, 7.5%, and 10% fucoidan incorporated against *Staphylococcus aureus* (*S. aureus*) and *Escherichia coli* (*E. coli*).

Treatment	Zone of Inhibition (mm)	
	*E. coli*	*S.aureus*
Gelatin	NI	NI*
Gelatin:Fucoidan 2.5%	8	5
Gelatin:Fucoidan 5%	10	7
Gelatin:Fucoidan 7.5%	10	8
Gelatin:Fucoidan 10%	12	10
Co‐amoxiclav	35	32
Gentamicin	17	12
Tetracycline	33	23

*Note*: The zone of inhibition is the average value of three replicates ± the standard deviation of the mean.

* No inhibition.

## 4 CONCLUSION

In this work, the effect of adding fucoidan on improving the properties of gelatin films was studied by physicochemical, antioxidant, and antimicrobial tests and an assay of microstructure and mechanical characteristics. Our results confirmed that composite gelatin–fucoidan film presented antibacterial effects and anti‐oxidative properties via donation of hydrogen atoms and scavenging free radicals. The gelatin‐fucoidan film also had mechanical impeccability with high tensile strength; in addition to barrier properties to be used in intermediate moisture foods. Films evaluated by FTIR and SEM analysis exhibited good interaction between gelatin and fucoidan to form a suitable composite film for innovative food applications.

## AUTHOR CONTRIBUTIONS

**Mohsen Pouralkhas:** Data curation (equal). **Moazemeh Kordjazi:** Investigation (equal); methodology (equal); project administration (equal); supervision (equal); writing – original draft (equal); writing – review and editing (equal). **Seyed Mahdi Ojagh:** Formal analysis (equal). **Omid Asadi Farsani:** Formal analysis (equal).

## CONFLICT OF INTEREST STATEMENT

The authors report no conflicts of interest.

## ETHICS STATEMENT

This study does not involve any human or animal testing.

## INFORMED CONSENT

Written informed consent was obtained from all study participants.

## Data Availability

Research data are not shared.
